# Bilateral synovial chondromatosis of the elbow in an adolescent: a case report and literature review

**DOI:** 10.1186/s12891-020-03322-1

**Published:** 2020-06-13

**Authors:** Jianming Mo, Jie Pan, Yun Liu, Wenyu Feng, Boxiang Li, Kai Luo, Weijia Mo, Huahao Lin, Shijie Liao

**Affiliations:** 1 Departments of Orthopedics, The First Affiliated Hospital of Guangxi Medical University, Nanning, 530021 Guangxi China; 2 Department of Rheumatology and Immunology, The First Affiliated Hospital of Guangxi Medical University, Nanning, Guangxi China; 3 Department of Guangxi, Medical University, Nanning, Guangxi China; 4 Departments of Pathology, The First Affiliated Hospital of Guangxi Medical University, Nanning, Guangxi China

**Keywords:** Synovial chondromatosis, Elbow joint, Bilateral, Adolescent, Athlete

## Abstract

**Background:**

Primary synovial chondromatosis is a rare benign disease that occurs in the joint mucosa.

**Case presentation:**

In this case report, a 14-year-old gymnast sustained pain in both elbows for 2 months with limited elbow joint activity. The initial diagnosis of bilateral elbow synovial chondromatosis was performed by physical examination and imaging report. Later, the patient was treated with open surgery on both sides of the elbow, including all loose bodies were removed out and the proliferative synovia were cut off. Histopathology reports confirmed synovial chondromatosis.

**Conclusions:**

The report introduced a case about synovial chondromatosis in bilateral elbow found in a 14-year-old girl, which is rarely involved in bilateral elbow and rarely found in adolescents. This case report aims to provide a treatment option for surgeons in similar situations.

## Background

Synovial chondromatosis is a benign tumor-like lesion of soft tissue cartilage such as joint synovium, which can lead to the formation of multiple cartilage nodules or loose bodies [1]. It usually occurs in 30s–50s, the incidence rate is 1 / 100,000, the male to female ratio is approximately 1.8: 1, but rarely found in children and adolescents [2, 3]. Synovial chondromatosis usually occurs unilaterally, rarely bilateral, in the large joints like the knee, but any joint could be involved, mostly affect large joints, such as knee, temporomandibular joints, hip, elbow, ankle and shoulder [1, 2]. Even in spinal cord, had been reported [4]. Moreover, synovial chondromatosis can be found intra-articular as well as extra-articular (like the extensor digitorum longus tendon), which were still relatively rare in the literature [5].

Although Henderson [6] reported the first elbow synovial chondromatosis in 1918, the etiology of synovial chondromatosis was currently uncertain. Bell et al. [7] believe that trauma is a risk factor for primary or secondary synovial chondropathy. The hypothesis regarding the disease was that loose bodies in the joint compartment did not have independent proliferating activity. The synovial membrane might be associated with the proliferation of the loose bodies by means of expressing cluster of differentiation 105 (CD105) and CD90 [8]. Moreover, relative studies had reported that fibroblast growth factor 2 (FGF-2) or transforming growth factor beta 3 (TGF-β3) were responsible for the formation of cartilaginous loose bodies and involved in the pathogenesis of synovial chondromatosis [9]. Milgram [10] summarized the clinical and pathological study of 30 cases of synovial chondromatosis, and concluded that there are three stages of development of the disease: stage I is synovial hyperplasia and hyperemia, synovial connective tissue cartilage metaplasia, but loose bodies has not appeared; stage II is active synovium Inflammation is associated with loose bodies; stage III is the regression of synovitis, with only a single or multiple loose bodies. Loose bodies were nourished by synovial fluid and have the ability to grow locally. If not treated early, it will cause irreversible damage to cartilage and joints, and can even be transformed into synovial chondrosarcoma [11]. Most loose bodies contain cartilage and its central bone or calcified components, as well as those with pure cartilage without bone, so simple radiography may not find them, and more sensitive computed tomography (CT) or Magnetic resonance imaging (MRI) is needed to help diagnose. Current treatments include drugs and surgery. For stage I, non-steroidal anti-inflammatory drugs can be used to relieve pain symptoms. For stage II and III, it is generally recommended to remove the diseased synovial membrane or remove the loose bodies [12]. Depending on the condition, the surgeon decides to use open or arthroscopic surgical resection.

Our case describes a 14-year-old female gymnast with bilateral elbow synovial chondropathy. To our knowledge, this is the first report of adolescent bilateral elbow joint synovial chondropathy, and the patient is a gymnast with a particular occupation.

## Case presentation

A 14-year-old girl, a gymnast, attended to our hospital with pain and restricted range of motion in her both elbows. Six years ago, she had an accident while training and presented with intermittent pain, without tissue swelling and limited range of motion of the elbow during that period. The elbow pain disappeared gradually after conservative treatment, mainly including rest, stop training and taking some non-steroidal anti-inflammatory drugs. Two years after, when the girl was on training, she suddenly felt pain on both the elbows and it kept on getting worse, with restriction of the elbow motion.

**Fig. 1:**
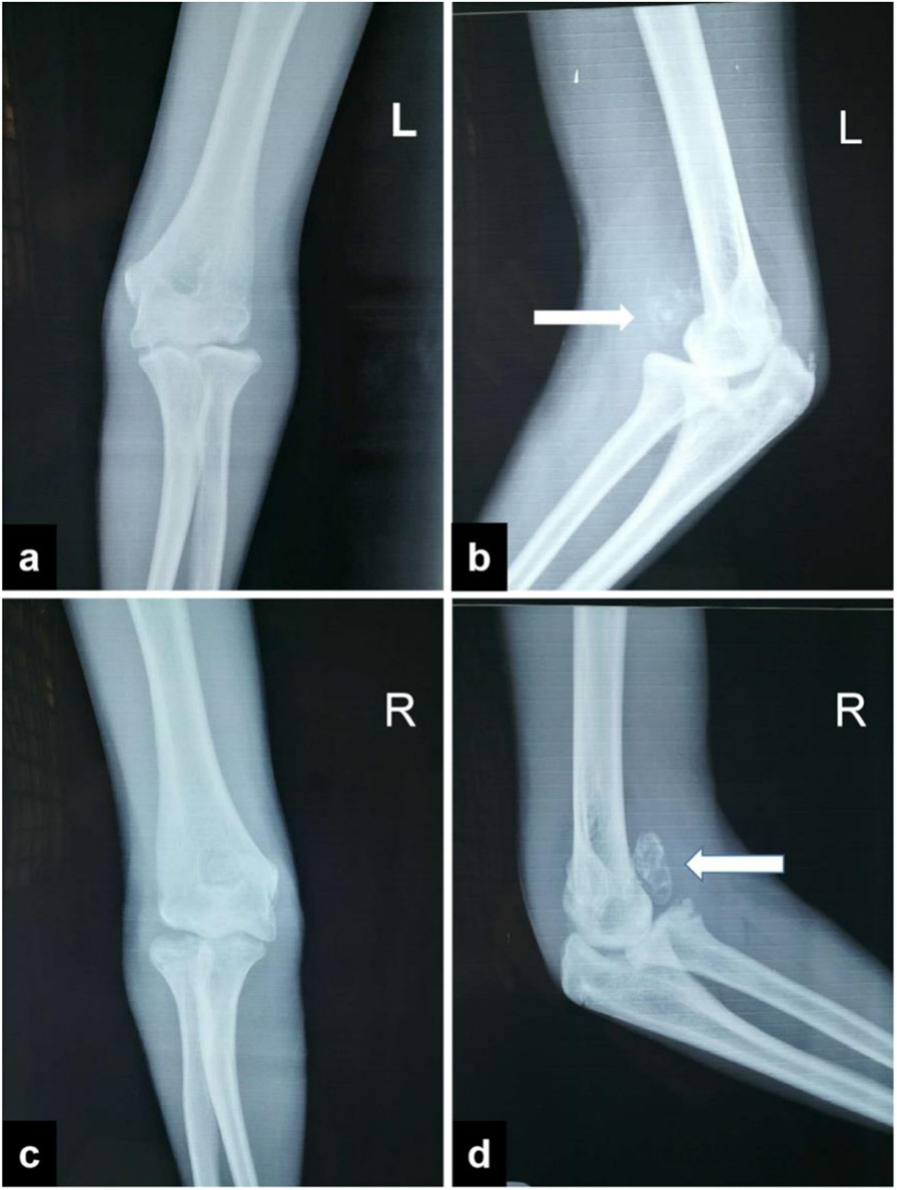
The initial X-ray (**a**, **c**, anteroposterior; **b**, **d**, lateral) of bilateral elbows showed calcified densities surrounding anterior and posterior aspects of the elbow joints. (**b**, **d** (arrow))

**Fig. 2:**
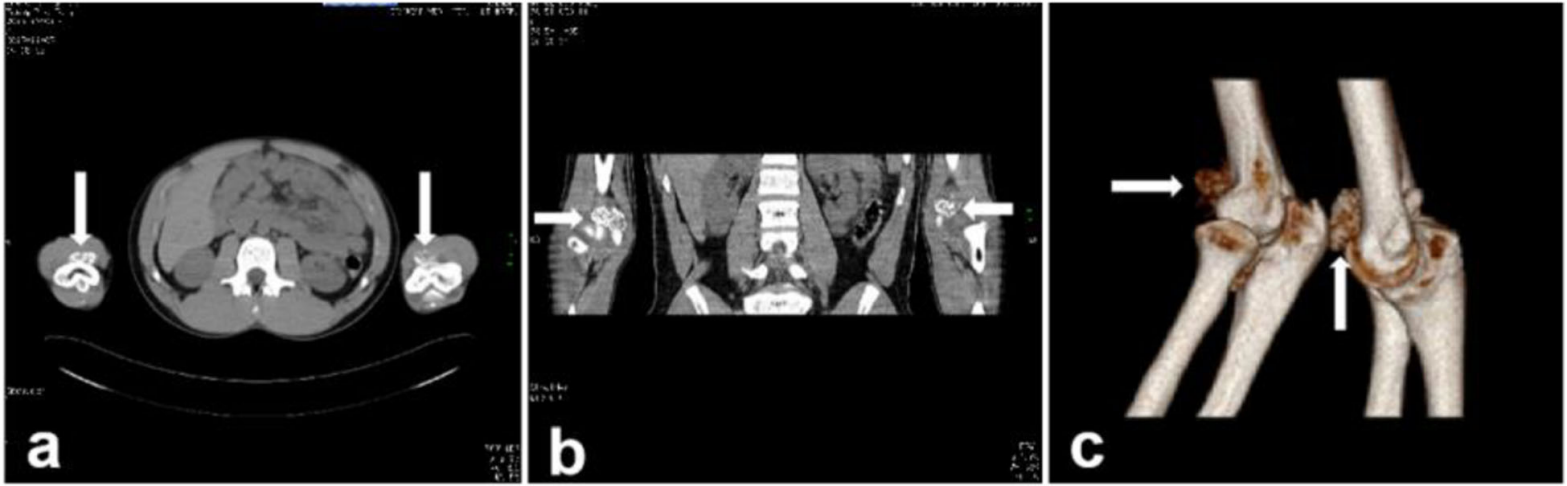
Computed tomography (CT) scan (**a**, transverse plane; **b**, coronal plane) and 3D-reconstruction (**c**) imaging of the bilateral elbows showed multiple calcified densities within the soft tissues surrounding anterior and posterior aspects of the elbow joints. Besides, there were sclerotic changes in the articular surface and the joints space were decreased. (arrow)

On physical examination, the significant increases in the size of both elbows were noticed. At palpation, masses were found in the bilateral elbow fossa. The left side was about 3.0 cm × 2.0 cm, and the right side was about 2.0 cm × 2.0 cm. The skin on the surface of the mass was free from redness, swelling and ulceration, and no venous bulging. Her elbow motion was poor on both sides, with 80 ° left flexion, 10 ° extension, right 90 ° flexion, 15 ° extension, so she could not touch her shoulders. However, the pronation and supination functions were not affected. Feelings and blood flow at the ends of the fingers of both upper limbs were normal. Laboratory tests were normal for white blood cell count, erythrocyte sedimentation rate, high-sensitivity C-reactive protein, and rheumatoid factor. Complementary imaging examinations and serum tests were performed and found no clinically considerable significance. The initial X-ray (Fig. 1) and CT and 3 Dimensions (3 D)-reconstruction imaging (Fig. 2) of the bilateral elbows showed multiple calcified densities surrounding anterior and posterior aspect of the elbow joints. Besides, there were sclerotic changes in the articular surface and the joints space were decreased in CT finding.

**Fig. 3:**
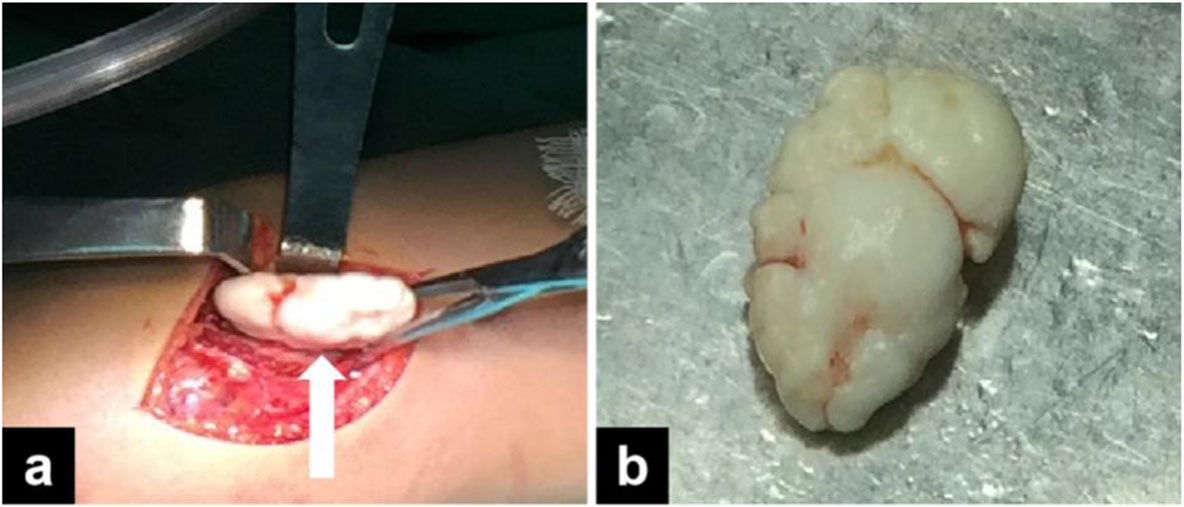
Appearance of loose bodies. **a** full exposure to general view during surgery.(arrow), **b** general view after resection

**Fig. 4:**
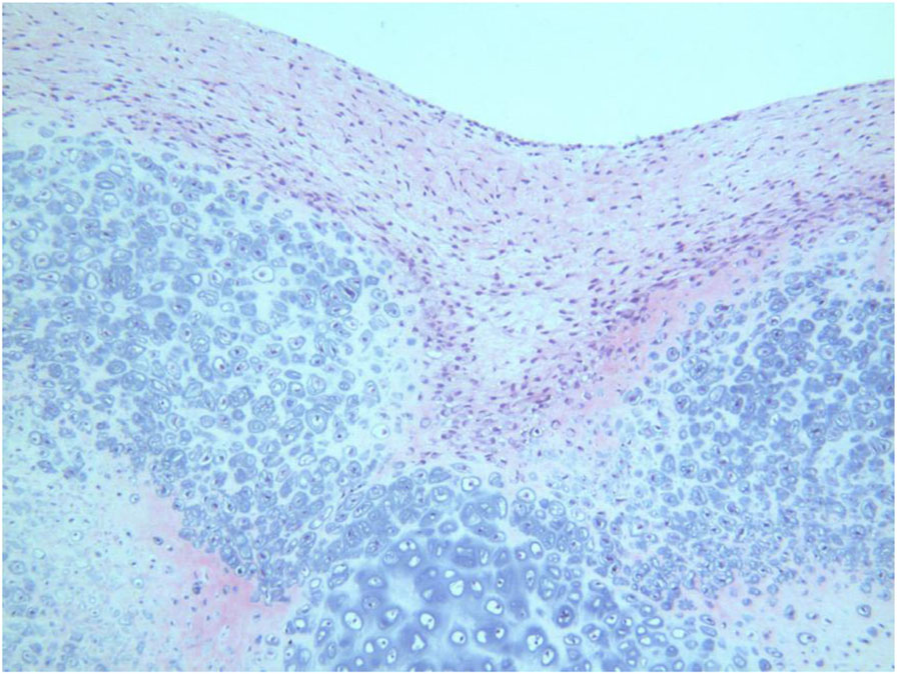
Osteochondral loose body, consisting of well-defined chondrocytes and mature cartilage tissues (Haematoxylin and Eosin stained,original magnification × 100)

Open surgery was performed simultaneously to the patient with diagnosis as Synovial Chondromatosis of bilateral elbow. First, cubital fossa was exposed and several loose bodies were observed anterior and posterior to the medial epicondyle in the joint capsule, varying in size from millimetres to 3 cm (Fig. 3). After Capsulotomy, all loose bodies were removed out and the proliferative synovia were cut off. Finally, loose bodies were collected for histological examination. Histological studies showed that chondrocytes and mature cartilage tissue formed nodular lesions, no abnormal cells or signs of malignancy were found, and the final diagnosis was synovial chondropathy. (Fig. 4).

**Fig. 5:**
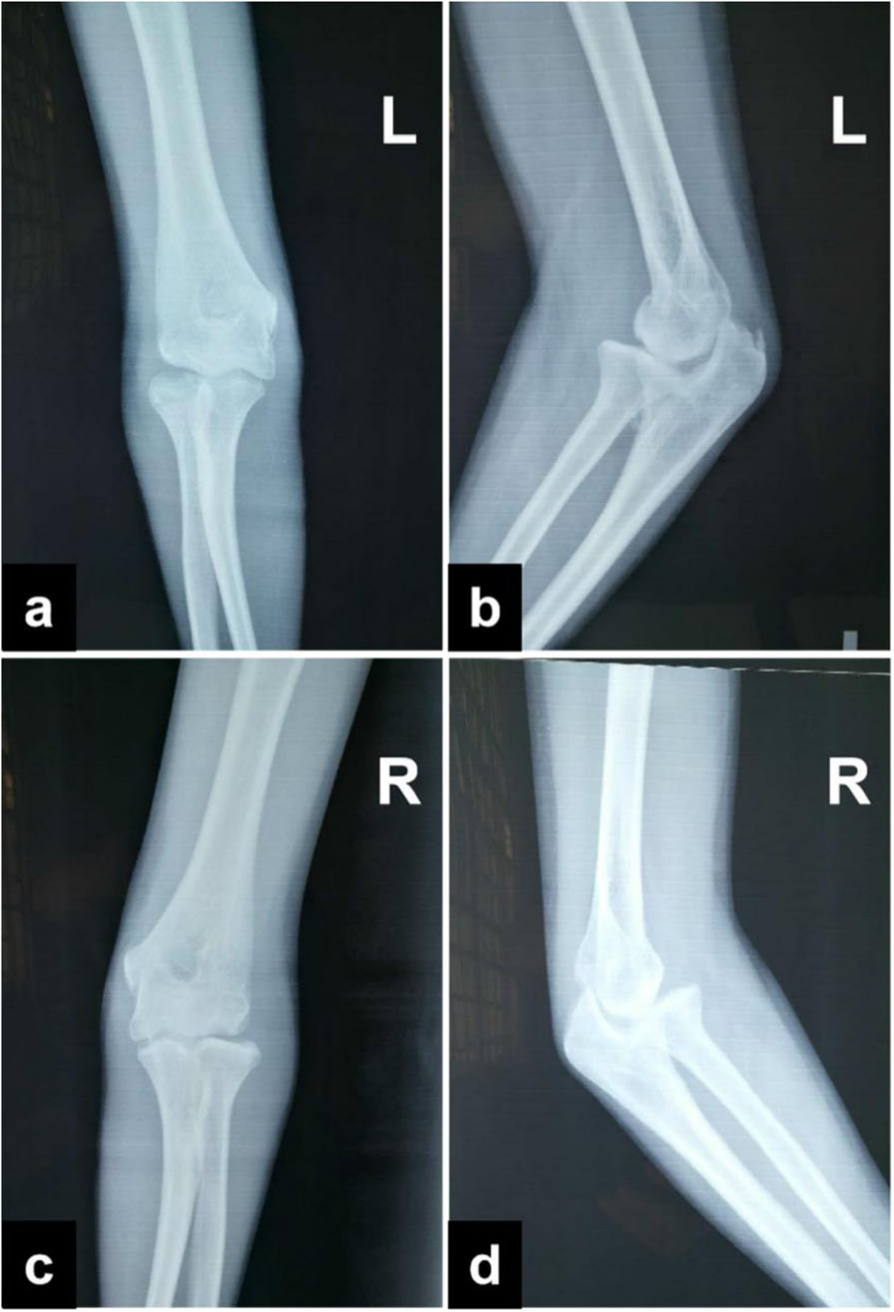
X-ray showing no calcific densities in bilateral elbow after operation

**Fig. 6:**
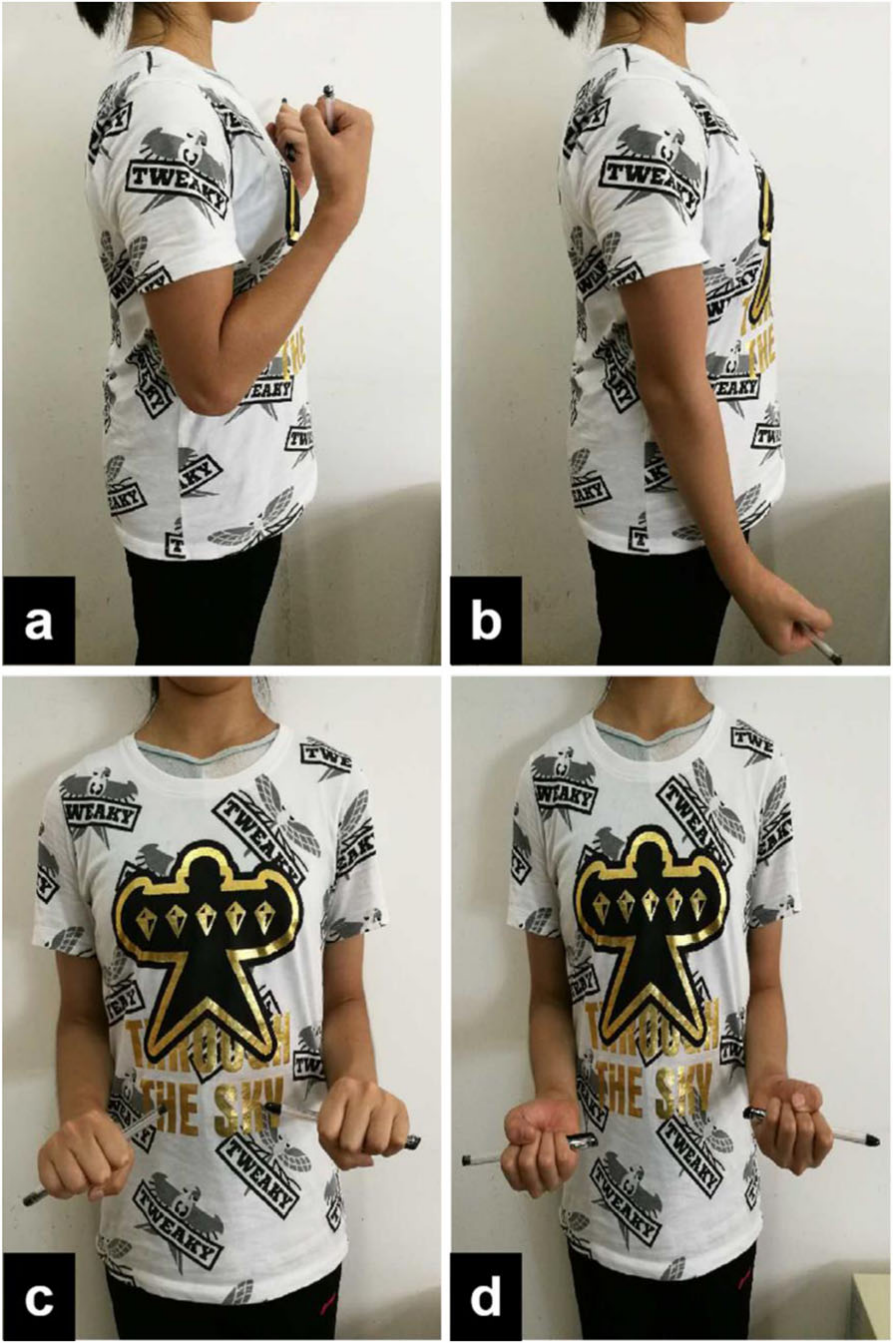
The photo of the elbow joint function 1 year after surgery showed that the flexion, extension, pronation and supination were not restricted

Postoperative elbow examination for passive movement was unrestricted and encourages active movement based on pain tolerance. Rehabilitation protocol: 1 ~ 3 d after surgery: Patients were educated to make them understand the significance of postoperative rehabilitation. Passively moving the affected shoulder and wrist joints to strengthen the isometric contraction of the affected limb muscles and active grasping of the affected hands to facilitate swelling. 4 d ~ 3w after surgery: passive flexion and extension of the wrist and stretching exercise, gradually doing biceps, triceps, wrist extension, wrist flexion, pronation, supination muscle group resistance exercise. Oral celebrex was given to patients within 3 weeks after surgery to reduce pain and prevent ectopic ossification. 4 ~ 8w after operation: continue to train the muscle strength of the affected limb and flexion and extension of the elbow. After each training, the affected elbow was applied with ice for 15 min. 2 ~ 6 months after operation: further strengthen the mobility training of the affected elbow joint, and gradually start the strength training of the affected limb and activity training of daily life. At a month after surgery the girl came for follow-up examination. She didn’t complain of any pain during that period and her range of motion were between − 10°and 130° in both the elbows. The X-ray was repeated during that period and found no calcified densities (Fig. 5). Her elbow joints were physical examined after 1 year, the flexion, extension, pronation and supination were not restricted.(Fig. 6).

## Discussion and conclusions

The clinical manifestation of synovial chondromatosis are comprising of pain, swelling, limited motion, palpable mass and locking of the elbow [1, 2]. Nerve compression, especially ulnar nerve, result in damaging sensory and motor of distal limbs. Because of the nonspecific symptoms and signs, it’s more challenging for clinician to distinguish from other diseases, such as synovial chondrosarcoma, pigmented villonodular synovitis (PVNS), osteochondritis dissecans, calcifying aponeurotic fibroma, elbow tuberculosis, hydroxyapatite deposition, and rheumatoid arthritis [13, 14].

Initial X-ray may show calcified nodules of various sizes and shapes on the elbow, and adjacent bones often have subchondral erosion or proliferative arthritis. CT owns its higher sensitivity than X-ray for the detection of ongoing calcification around or within loose bodies. MRI is important for diagnosis. If the patient can afford it, an MRI is recommended for each patient. MRI can accurately reflect the pathological changes of synovial chondromatosis and clear the scope of the lesion, and can also identify the adjacent soft tissue, bone marrow and neurovascular involvement. It provides invaluable information to the clinician regarding the need of conservative or surgical therapy in those patients suffering from diseases [1]. Pathological examination is the gold standard technique to make the diagnosis.

Drugs and surgery are commonly used treatments for synovial chondromatosis. Partial pain can often be relieved by non-steroidal anti-inflammatory drugs. However, Surgical treatment is more advocated in removal of loose bodies and synovectomy should be done for the prevention of recurrence and delay the secondary osteoarthritis [1]. Clinician prefer to arthroscopic operation at the present time because of good visualisation, more safety and effectiveness, less trauma, lower morbidity, and the permission of early rehabilitation [1]. However, there are no published data comparing the results of the open and arthroscopic surgery**.** In our case, since lacking of the evidence to distinct whether all loose bodies were intra-articular or not, we chose the open method to get more excellent exposure and better chances for disease eradication [15].

Generally, disease is self-limiting. However, recurrence, usually results from the incomplete removal of the loose bodies or diseased synovium at the initial surgery. Zhu et al. [12] reported 11 cases of articular endoscopic treatment of elbow synovial chondromatosis, and found a recurrence rate of 18.2% (2/11). Bell et al. [7] reported a case of temporomandibular joint synovial chondropathy. Due to concerns about damaging deep tissues, the tumor was not removed, but after 18 months of observation, no signs of recurrence were found. It is speculated that continuous trauma after surgery may be the risk factor for relapse. Aydin et al. [16] Who reported the same cases as Bell, considered that metaplastic activity during treatment was the main risk factor for relapse. Malignant degeneration into chondrosarcoma had also been reported, although this transformation is rare [1]. *Evans* found that 5 of 78 cases of primary synovial chondromatosis had a chondrosarcoma transition [17]. McCarthy et al. [18] studied 155 cases of primary synovial chondromatosis and found 4 cases of malignant transformation. Thus, early surgical treatment and regular follow-up are necessary to improve the prognosis for the disease.

The report introduced a case about synovial chondromatosis in bilateral elbow found in a 14-year-old girl, which is rarely involved in bilateral elbow and rarely found in adolescents. The patient of the case was a gymnast where range of motion was highly required. The open surgery was done to completely remove the loose bodies. During the follow-up, The patient wentback to training 1 month later without restricted range of motion and pain in her elbows. So far, patients still have no elbow pain and limited mobility. Postoperative imaging examination showed no signs of recurrence.

## Data Availability

All the data needed to achieve the conclusion are presented in the paper.

## Abbreviations

CT: Computed Tomography
3D: 3 Dimensions
CD: Cluster of Differentiation
FGF-2: Fibroblast Growth Factor 2
TGF-β3: Transforming Growth Factor Beta 3
PVNS: Pigmented Villonodular Synovitis
MRI: Magnetic resonance imaging
